# Study on the Robust Bridge Deck Pavement System Based on Horseshoe-Shaped Shear Keys

**DOI:** 10.3390/ma18051095

**Published:** 2025-02-28

**Authors:** Sheng Li, Hanglin Luo, Yichen Zhao, Xiaojun Zhou

**Affiliations:** 1 Department of Road and Bridge Engineering, Sichuan Vocational and Technical College of Communications, Chengdu 611130, China; 13541361637@163.com (S.L.);; 2 School of Architecture and Civil Engineering, Xihua University, Chengdu 610039, China

**Keywords:** bridge, horseshoe-shaped shear key, concrete, interfacial bonding agent, fiber, mechanical properties

## Abstract

Traditional concrete bridge decks often incorporate steel mesh to ensure connection and prevent cracking. However, the cracking in the connecting layer, low bond strength, misalignment of steel mesh, and settling at the bottom often appear. In this study, fiber-reinforced concrete was used for the bridge deck overlay, and a horseshoe-shaped shear key was employed to connect it with the beam body, forming a robust composite bridge deck system. By optimizing the concrete composition and interface bonding methods within the system, a comprehensive investigation was conducted into the compressive and splitting tensile strengths of different composite systems. The findings showed that the horseshoe-shaped shear key enhances the splitting tensile strength of the composite structural system while maintaining its compressive strength, ensuring a certain level of structural integrity during failure. As the strength grade of the steel fiber-reinforced concrete in the deck overlay increases, the compressive and splitting tensile strengths of the composite system initially rise and then stabilize, with C40 being the optimal strength grade for the deck overlay concrete. Furthermore, the overall performance of the deck overlay concrete with steel fibers is superior to that with the POM and PP fibers. The application of the YJ-302 interface bonding agent at the connection between the deck overlay and the beam body concrete further enhances the mechanical properties of the composite system.

## 1. Introduction

**Figure 1:**
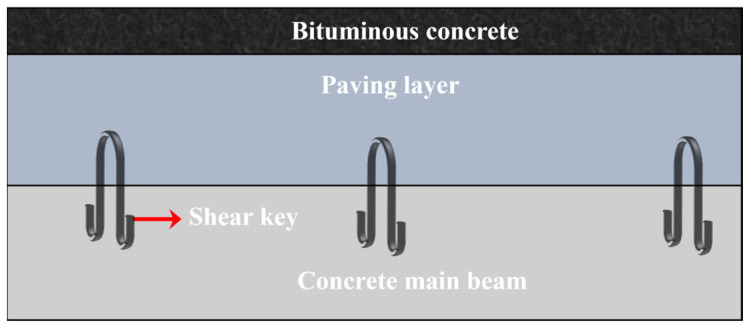
The type of bridge in this study.

The bridge superstructure usually consists of a concrete beam at the bottom, a concrete paving layer in the middle, and an asphalt pavement at the top, which is also the type of bridge in this study, as Figure 1 shows. As a vital component of the bridge superstructure, bridge deck pavement is laid on top of the bridge deck and directly bears vehicular loads. Consequently, the materials used for the bridge deck pavement should exhibit high strength, good toughness, and resistance to cracking. Cement and concrete are the primary materials for bridge deck pavement due to boasting advantages, such as high compressive strength [1,2,3,4], significant stiffness [5], excellent abrasion resistance [6,7], and low costs [8,9,10]. However, issues like thermal stress, autogenous shrinkage, and drying shrinkage in cement and concrete can lead to cracking risks [11,12,13], thereby affecting the bonding performance and integrity of the bridge superstructure. Furthermore, when the bridge deck is subjected to substantial loads, significant shear stresses arise within the pavement layer. If the shear resistance between the pavement layer and the bridge deck is weak [14,15], it can result in slippage, debonding, and other defects, severely impacting the durability of the pavement structure [16,17,18]. Therefore, enhancing the interlayer bonding performance between the beam and the pavement concrete is of importance.

Afandi et al. [19] suggested that the primary factors influencing the interfacial bonding performance between the concrete layers are the surface treatment methods of the concrete and the concrete properties. In practical engineering, interface treatments such as scarification [20,21], grooving [22,23], and sandblasting [24,25] are commonly employed to improve the interfacial bonding performance of bridge deck pavements. These methods primarily increase the roughness of the concrete surface, thereby enhancing the interfacial shear resistance. However, controlling the outcomes of interface treatments to enhance interlayer bonding performance in concrete remains challenging. Excessive disturbance of the interface can lead to crack formation on the bridge deck, reducing interlayer bonding performance, while insufficient treatment fails to provide adequate roughness [26]. With the gradual advancement in concrete technology, fiber-reinforced concrete has been widely used due to its superior tensile strength. Studies have shown that incorporating steel fibers or other fibers into concrete can enhance its crack resistance and abrasion resistance [27,28,29,30], making it suitable for infrastructure projects like roadways and bridges. Kim and Bordelon [31] investigated the influence of fiber-reinforced mortar on the interfacial bonding of old concrete and found that bond specimens with fiber-reinforced mortar exhibited higher splitting tensile strength than those without fibers. Evidently, fibers have a positive impact on enhancing the interlayer bonding performance of concrete. However, excessive vehicle load and impact load would still cause an interlayer slip of the bridge floor structure. Therefore, a single interlayer bonding enhancement measure cannot completely prevent the occurrence of cracking or sliding phenomenon of interlayer structure.

Furthermore, enhancing the interlayer bonding performance of concrete can also be achieved through methods such as spraying interfacial bonding materials on the surface [32,33] and increasing anchoring reinforcement [34]. Magnesium Phosphate Cement (MPC) is a novel cementitious material composed of magnesium oxide (MgO) and phosphate [35,36,37], rapidly reacts with water to form magnesium ammonium phosphate hexahydrate (MgNH_4_PO_4_·6H_2_O) or magnesium potassium phosphate hexahydrate (MgKPO2084·6H_2_O) [38], along with the development of its mechanical properties. The MPC is characterized by rapid hardening, strong bonding capabilities, and low drying shrinkage [39,40], making it increasingly popular in repair materials. Li et al. [41] demonstrated that when MPC is used as a repair material, the repaired specimens exhibit excellent bonding performance, enhancing the mechanical properties of the specimens. However, the application of magnesium phosphate cement in practical engineering is limited by its poor water resistance, fast setting time, and high cost. Polymer binders also provide good bonding effects [42]. In addition to using bonding materials, Seible et al. [43] and others have significantly improved the shear capacity between the pavement layer and the bridge deck by roughening the bonding surface of the bridge and arranging anchoring reinforcement at the interface, effectively controlling interlayer slippage.

**Figure 2:**
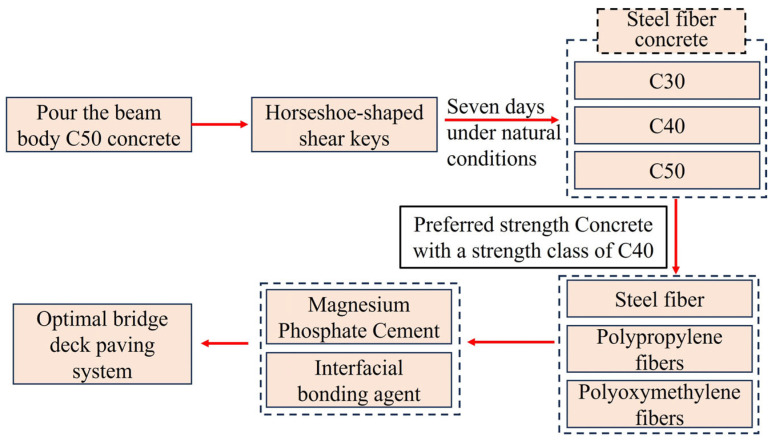
The main process of this study.

As such, current efforts to enhance the bonding performance between the bridge decks and pavement layers primarily focus on four aspects: modifying concrete properties, increasing interface roughness, utilizing interfacial bonding materials, and adding anchoring reinforcement. However, most studies have only tested a single method and have not explored the coupled effects of multiple methods. Therefore, it is of great practical significance to study the coupling effect of various interfacial consolidation forms for optimizing the interlayer structure of bridge deck pavement. This experiment primarily investigated the influence of factors such as the type of fiber-reinforced concrete in the pavement layer, horseshoe-shaped shear keys, and interfacial bonding materials (polymer binder, MPC) on the bonding performance between the bridge deck pavement layer and the beam. The mechanical properties of the composite system are evaluated through the compressive strength and splitting tensile strength tests, and the microstructure is utilized to explore the mechanisms underlying the interfacial bonding performance. The main process of this study is shown in Figure 2. The purpose of this study is to increase the bond strength of bridge deck pavement structure and reduce the slip of bridge deck pavement in practical engineering.

## 2. Materials and Methods

### 2.1. Raw Materials

**Figure 3:**
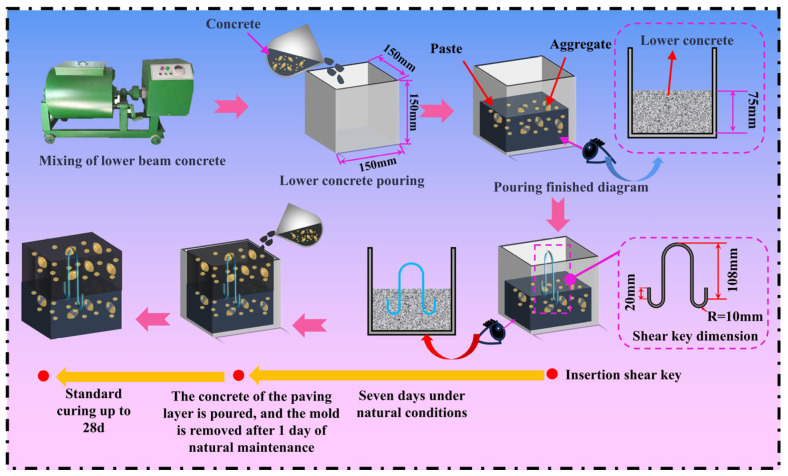
Schematic diagram of composite structure pouring.

The cement used in the experiment was P·O 42.5R cement provided by Hongshi Cement Co., Ltd. in Changning County, Sichuan Province, China. The specific technical indicators are shown in Table 1. Grade II fly ash was selected for use. Silica fume was supplied by Chengdu Keliang Building Materials Co., Ltd. (Chengdu, China). The coarse aggregate consisted of continuously graded crushed stone ranging from 5 to 20 mm. The fine aggregate was manufactured sand with a fineness modulus of 2.9 and a stone powder content of 5.0%. The superplasticizer used was GX-20, with a water reduction rate of 25.4%. Additionally, the fibers used in the pavement layer concrete included steel fibers (SF), polypropylene fibers (PP), and polyoxymethylene fibers (POM). The bonding materials employed were Magnesium Phosphate Cement (MPC) and YJ-302 concrete interfacial bonding agent (IBA). The shear keys used in the experiment were horseshoe-shaped shear keys (HSSK) made from 8 mm diameter plain round steel bars, with the shape and parameters of the horseshoe-shaped shear keys shown in Figure 3. The mix proportions used are presented in Table 2.

### 2.2. Sample Preparation and Test Methods

Using the prepared concrete mix proportions and the HSSKs, 150 × 150 × 150 mm cubic composite structural systems were fabricated, both with and without HSSKs. These systems were designed to investigate the effects of horseshoe-shaped steel bars, the strength grade of the pavement layer concrete, different fiber types in the pavement layer concrete, and various bonding agents between the beam body and the pavement layer concrete on the compressive strength and splitting tensile strength of the composite structure.

The fabrication process of the composite systems was divided into three main steps. Firstly, C50 beam body concrete was poured into the mold and vibrated, with a pouring height of half the mold (75 mm). Secondly, HSSK was inserted (or not inserted) into the vibrated beam body concrete. The mold was then covered with a thin film and cured in a standard curing box for 7 days. Thirdly, pavement layer concrete of different strength grades or containing different fibers was poured into the mold, vibrated to ensure compaction, and cured in a standard curing box. A schematic diagram of the pouring process for the composite structure is shown in Figure 3. Additionally, to explore the impact of bonding agents on the mechanical properties of the composite system, the bonding agent was applied to the surface of the beam body concrete immediately before pouring the pavement layer concrete.

**Figure 4:**
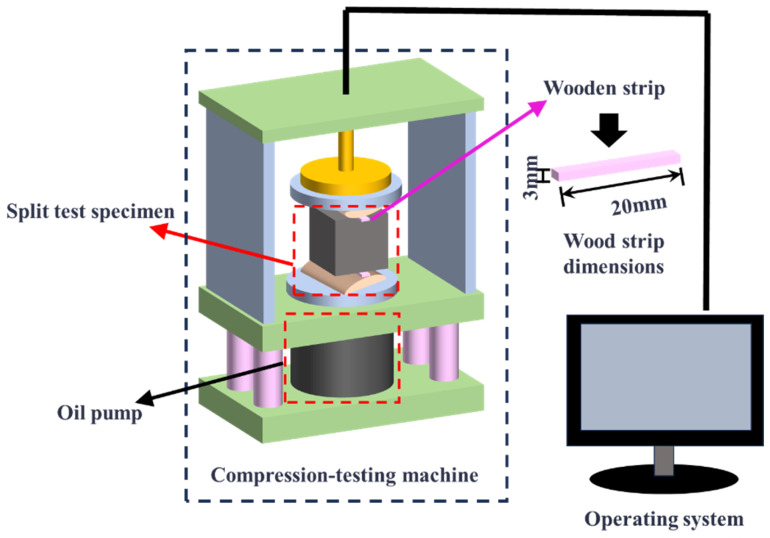
Schematic diagram of splitting tensile test operation.

The tests for compressive strength and splitting tensile strength of the concrete were conducted in accordance with the test methods specified in the “Standard for test methods of physical and mechanical properties of concrete” (GB/T 50081-2009). The specimens were molded and cured to an age of 28 days before testing their compressive and splitting tensile strengths. The splitting tensile strength test of concrete is shown in Figure 4. Microscopic testing and analysis of the samples were performed using scanning electron microscopy to investigate the mechanism of the influence of the interfacial bonding agent and fibers on the composite system.

## 3. Experimental Results and Discussions

### 3.1. Effect of Pavement Concrete Strength Grade

#### 3.1.1. Compressive Strength

**Figure 5:**
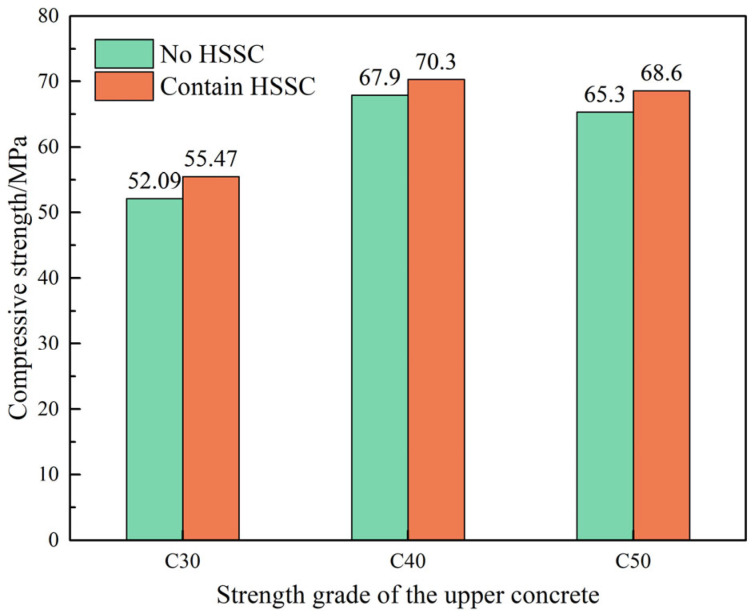
Influence of strength grade of steel fiber reinforced concrete on compressive strength of composite structure system.

The influence of steel fiber-reinforced pavement layer concrete with varying strength grades, combined with HSSKs, on the compressive strength of the composite system is illustrated in Figure 5. The figure reveals that, regardless of the presence of the HSSK, the compressive strength of the composite system initially increases and then decreases as the strength grade of the pavement layer concrete rises. This indicates that the optimal compressive strength of the composite system is achieved when the strength of the pavement layer concrete is relatively close to that of the beam body concrete. When the strength difference between upper and lower concrete is large, the concrete of the lower strength will be destroyed first. Additionally, in the presence of the HSSK, the compressive strength of the composite system experiences a slight enhancement, albeit with a limited increase in magnitude. From the perspective of compressive strength, the optimal combination for the composite system is HSSK paired with C40 steel fiber-reinforced pavement layer concrete.

#### 3.1.2. Splitting Tensile Strength

**Figure 6:**
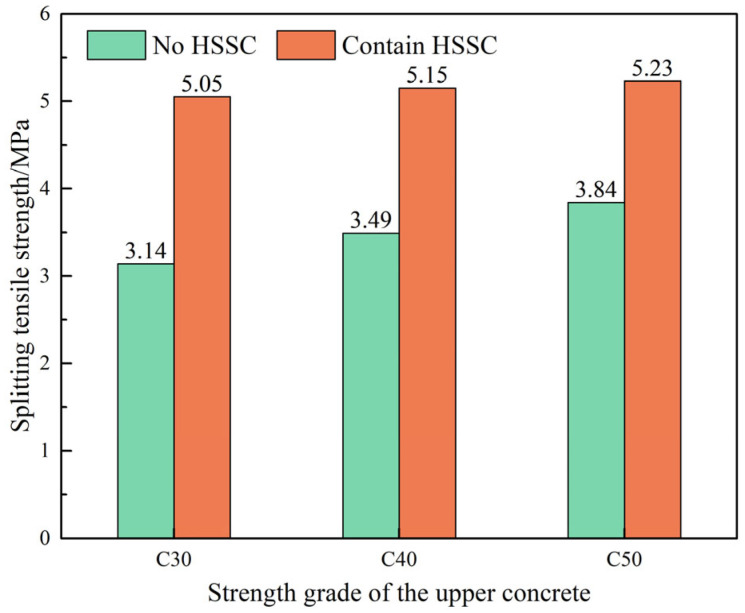
Influence of different strength grades of steel fiber reinforced concrete on the splitting tensile strength of the composite structural system.

The effect of steel fiber-reinforced pavement layer concrete with different strength grades, in conjunction with HSSKs, on the splitting tensile strength of the composite system is depicted in Figure 6. The figure shows that, in the absence of HSSK, the splitting tensile strength of the composite system gradually increases with the elevation of the pavement layer concrete strength grade. Specifically, when the strength grades of the pavement layer concrete are C30, C40, and C50, the corresponding splitting tensile strengths of the composite system are 3.14 MPa, 3.49 MPa, and 3.84 MPa, respectively. In the presence of the shear connectors, the splitting tensile strength of the composite system exhibits a slight increase as the strength grade of the pavement layer concrete rises, with strengths of 5.05 MPa, 5.15 MPa, and 5.23 MPa for C30, C40, and C50, respectively. These results suggest that in the absence of HSSK, the splitting tensile strength of the composite system is primarily influenced by the strength grade of the pavement layer concrete. However, when the HSSKs are present, the impact of the pavement layer concrete strength grade on the splitting tensile strength of the composite system is relatively minor. Therefore, from the perspective of splitting tensile strength, a C30 strength grade for the pavement layer concrete is acceptable. Nonetheless, considering both compressive strength and splitting tensile strength, the optimal combination for the composite system is horseshoe-shaped shear connectors paired with C40 steel fiber-reinforced pavement layer concrete.

**Figure 7:**
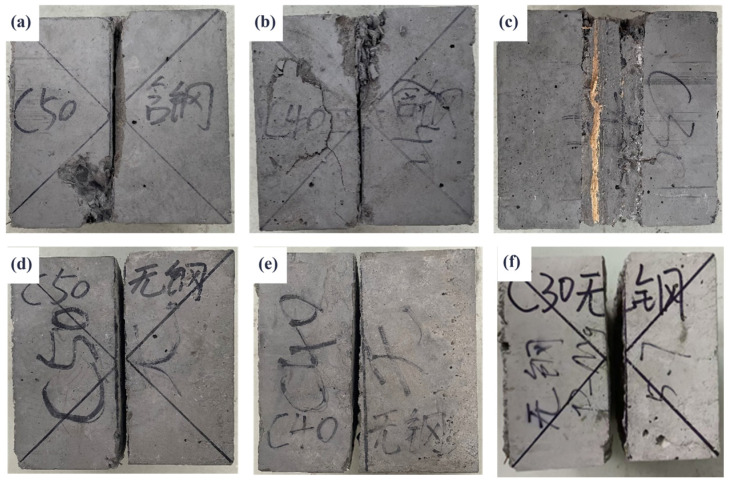
Failure modes of different strength grade pavement layer concrete + shear bond system in splitting tensile test. (**a**) C50 paving layer + HSSK; (**b**) C40 paving layer + HSSK; (**c**) C30 paving layer + HSSK; (**d**) C50 paving layer and no HSSK; (**e**) C40 paving layer and no HSSK; (**f**) C30 paving layer and no HSSK.

**Figure 8:**
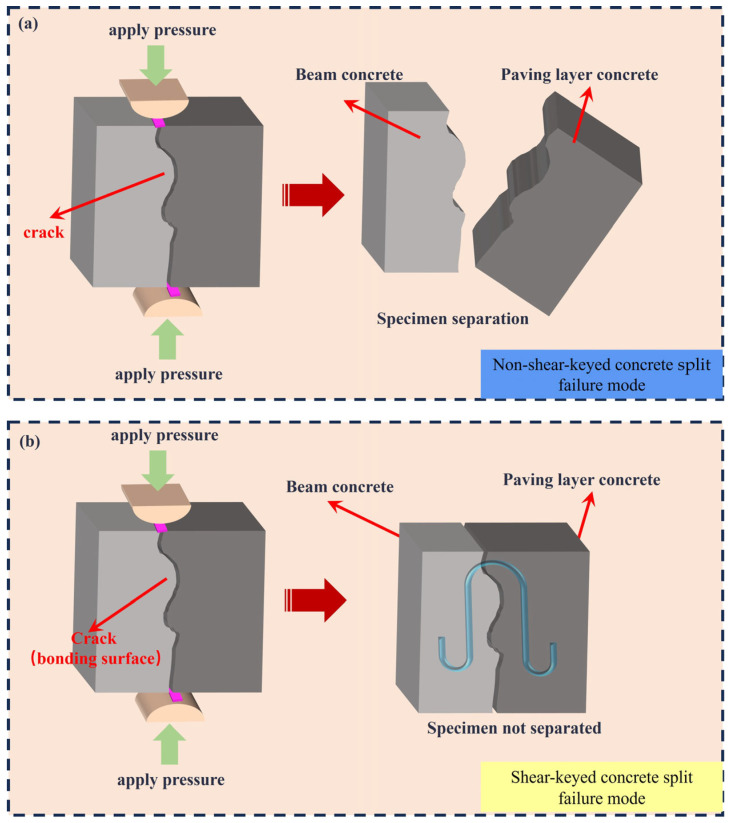
Schematic diagram of split failure of composite structure. (**a**) within HSSK; (**b**) no HSSK.

The failure modes of the splitting tensile tests for the composite systems, consisting of steel fiber-reinforced pavement layer concrete with varying strength grades and HSSKs, are illustrated in Figure 7. Figure 7a–c demonstrate that, in the absence of HSSKs, all the concrete samples exhibit splitting failure at the bonding interface, resulting in the samples being split into two halves. In contrast, as shown in Figure 7d–f, the incorporation of HSSKs results in the primary failure plane still occurring at the bonding interface, but the samples maintain their integrity upon final failure. This phenomenon indicates that HSSKs can enhance the integrity of the samples during the splitting failure process. Additionally, the tensile strength at the bonding interface of the composite system is the lowest, making it the weakest interface during the splitting failure process. The splitting failure diagram of the combined structure is shown in Figure 8.

### 3.2. Effect of Pavement Concrete Fiber Type

#### 3.2.1. Compressive Strength

**Figure 9:**
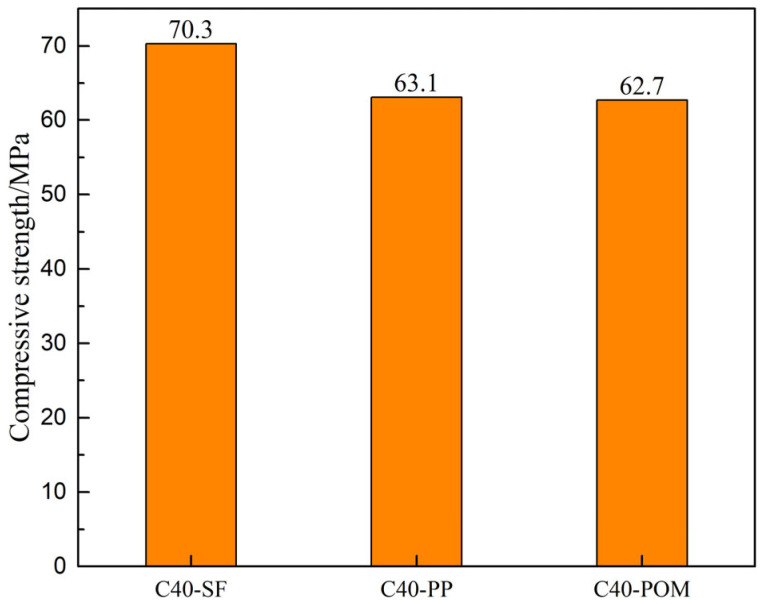
Influence of fiber types in pavement layer concrete on compressive strength of composite structure system.

With the strength grade of the pavement layer concrete fixed at C40, an investigation was conducted to explore the influence of varying fiber types within the pavement layer concrete, alongside the HSSK, on the compressive strength of the composite system. The results are presented in Figure 9. As illustrated, the composite system exhibits the highest compressive strength when the pavement layer concrete contains steel fibers. Conversely, the compressive strength of the system decreases when the pavement layer concrete incorporates PP fibers or POM fibers.

#### 3.2.2. Splitting Tensile Strength

**Figure 10:**
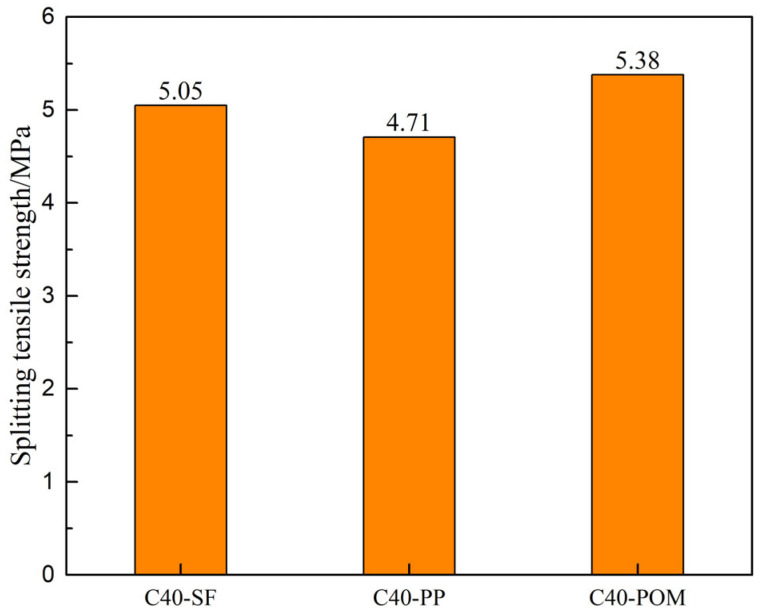
Influence of fiber types in pavement layer concrete on the splitting tensile strength of the composite structural system.

Maintaining the strength grade of the pavement layer concrete at C40, an exploration was undertaken to assess the impact of different fiber types in the pavement layer concrete, combined with HSSK reinforcement, on the splitting tensile strength of the composite system. The findings are depicted in Figure 10. The results indicate that the composite system achieves the highest splitting tensile strength when the pavement layer concrete contains POM fibers, while the lowest strength is observed with PP fibers. Nevertheless, the splitting tensile strength of the composite system remains above 4.7 MPa for all three fiber types, indicating robust toughness across the board.

**Figure 11:**
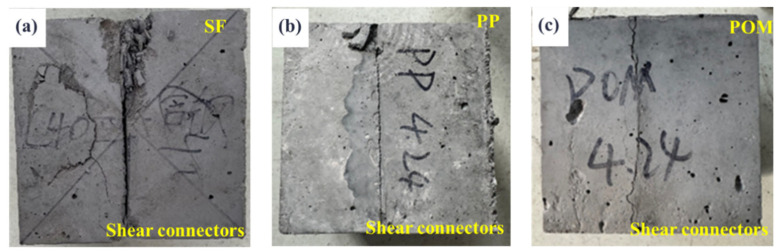
Failure modes of the composite structure system in the splitting tensile test when the pavement layer contains different fibers: (**a**) SF; (**b**) PP; (**c**) POM.

**Figure 12:**
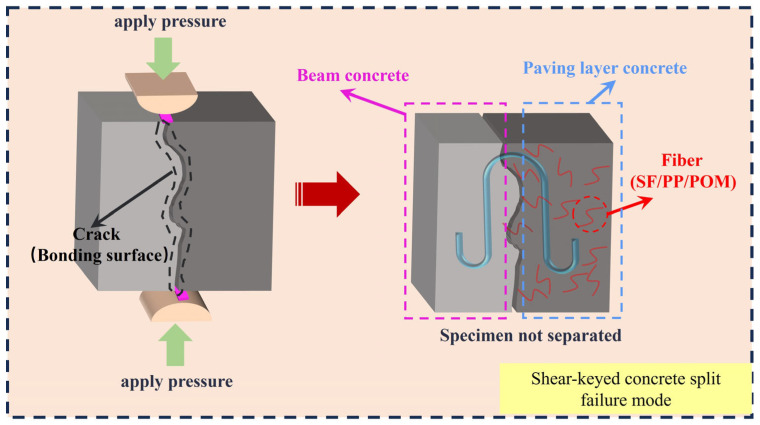
Schematic diagram of the split failure of the combined structure with HSSK and fiber.

Figure 11 presents the failure modes of samples after the splitting tensile tests conducted on the composite system with pavement layer concrete containing various fiber types and HSCCs. The failure patterns observed are nearly consistent across different fiber types, primarily characterized by splitting failure at the bonding interface. However, no significant separation between the pavement layer concrete and the beam body concrete is evident, highlighting the excellent coherence of the composite system. Figure 12 shows the schematic diagram of the split failure of the combined structure with HSSK and fiber.

#### 3.2.3. Microstructure

**Figure 13:**
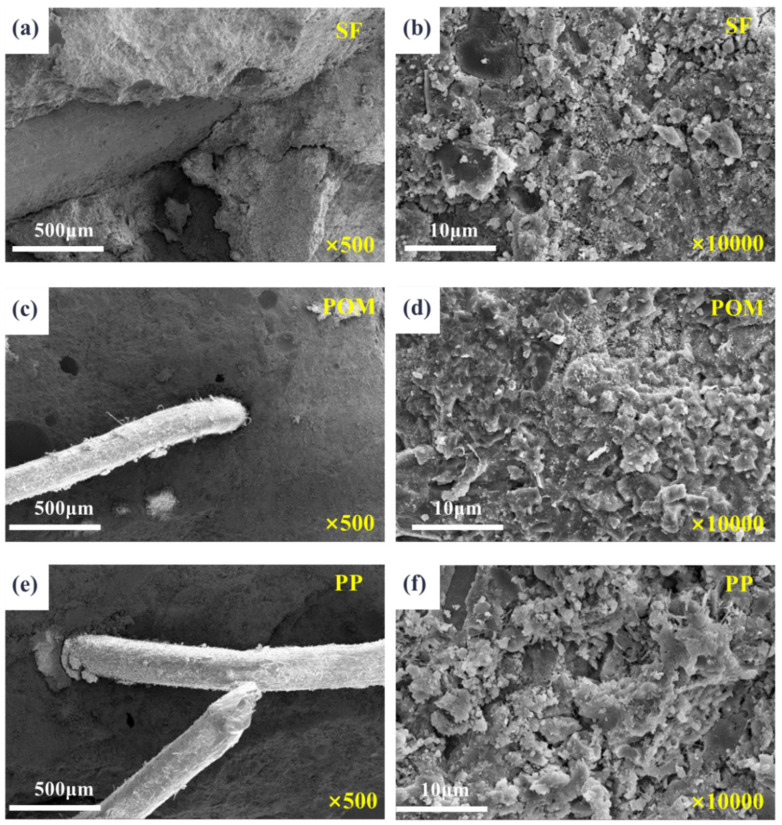
Microstructure of pavement concrete with different fibers. (**a**) SF fiber + 500×; (**b**) SF fiber + 10,000×; (**c**) POM fiber + 500×; (**d**) POM fiber + 10,000×; (**e**) PP fiber + 500×; (**f**) PP fiber + 10,000×.

The microstructures of pavement layer concrete with different fiber types are illustrated in Figure 13. The images reveal that the incorporation of various fibers has minimal impact on the microstructure and composition of the pavement layer concrete, resulting in relatively small variations in the overall compressive strength of the composite system. Notably, steel fibers are the most effectively embedded within the mortar, causing the least disruption to its density. Consequently, the steel fibers exert the most significant influence on the comprehensive mechanical properties of the composite system.

### 3.3. Effect of Interfacial Bonding Agent

#### 3.3.1. Compressive Strength

**Figure 14:**
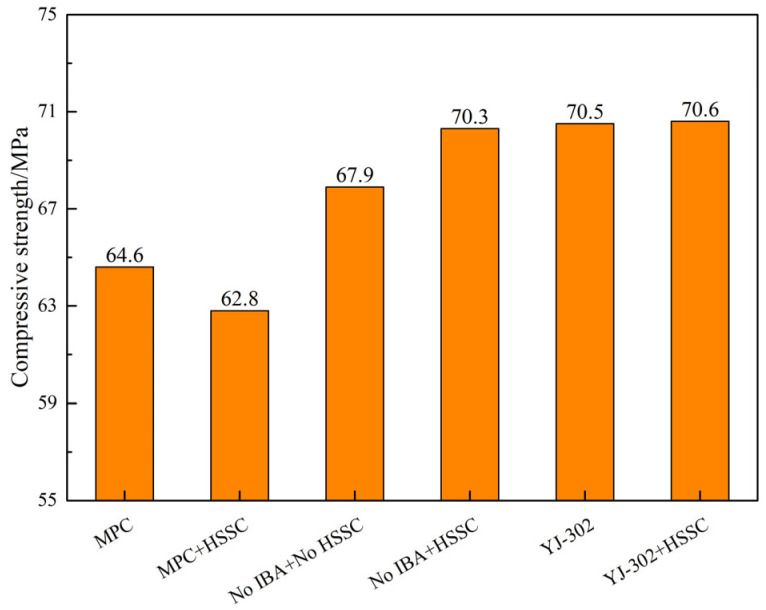
Influence of binder and horseshoe shear bond on compressive strength of composite structure system.

With the strength grade and fiber type of the pavement layer concrete fixed at C40 and steel fibers, an investigation was conducted to explore the influence of the bonding agents and HSSKs on the mechanical properties of the composite system. The test results for compressive strength of different composite systems are presented in Figure 14. As shown, the composite system without a bonding agent and HSSKs exhibits a compressive strength of 67.9 MPa. Upon the addition of HSSK, this value increases to 70.3 MPa. However, the incorporation of magnesium phosphate cement (MPC) into the system results in a decrease in strength, with values of 62.8 MPa and 64.6 MPa for systems with and without HSSKs, respectively. This reduction is attributed to the rapid setting time of MPC, which leads to a loss of workability within minutes, making the construction difficult and compromising the interface bonding effect. Consequently, a weak interface is introduced between the beam body concrete and the pavement layer concrete, leading to strength reduction. Nevertheless, the composite system with an interface bonding agent demonstrates a strength value close to that of the system with only HSSKs, indicating the excellent bonding performance of YJ-302 and suggesting that the bonding agent alone does not further enhance the compressive strength of the composite system.

#### 3.3.2. Splitting Tensile Strength

**Figure 15:**
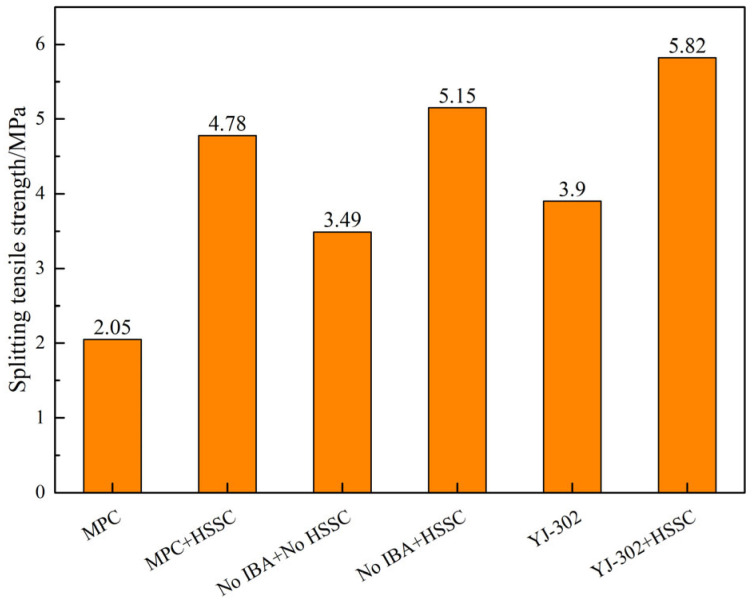
Influence of binder and horseshoe shear bond on the splitting tensile strength of the composite structural system.

The test results for splitting tensile strength of different composite systems are shown in Figure 15. In the absence of a bonding agent and HSSKs, the composite system exhibits a splitting tensile strength of 3.49 MPa. The application of the YJ-302 interface bonding agent increases this strength to 3.90 MPa, demonstrating its ability to enhance the splitting tensile strength of the composite system. Conversely, the use of MPC as a bonding agent results in a decrease in splitting tensile strength to 2.05 MPa due to its rapid setting time and the associated construction difficulties that compromise the bonding effect. Additionally, the incorporation of HSSKs significantly improves the splitting tensile strength of the composite system, regardless of the presence of a bonding agent. In the presence of HSSKs, the splitting tensile strengths of systems with MPC, no bonding agent, and YJ-302 interface bonding agent are 4.78 MPa, 5.15 MPa, and 5.82 MPa, respectively. Collectively, these results indicate that the combination of HSSKs and YJ-302 interface bonding agent yields the optimal compressive and splitting tensile strengths for the composite system.

**Figure 16:**
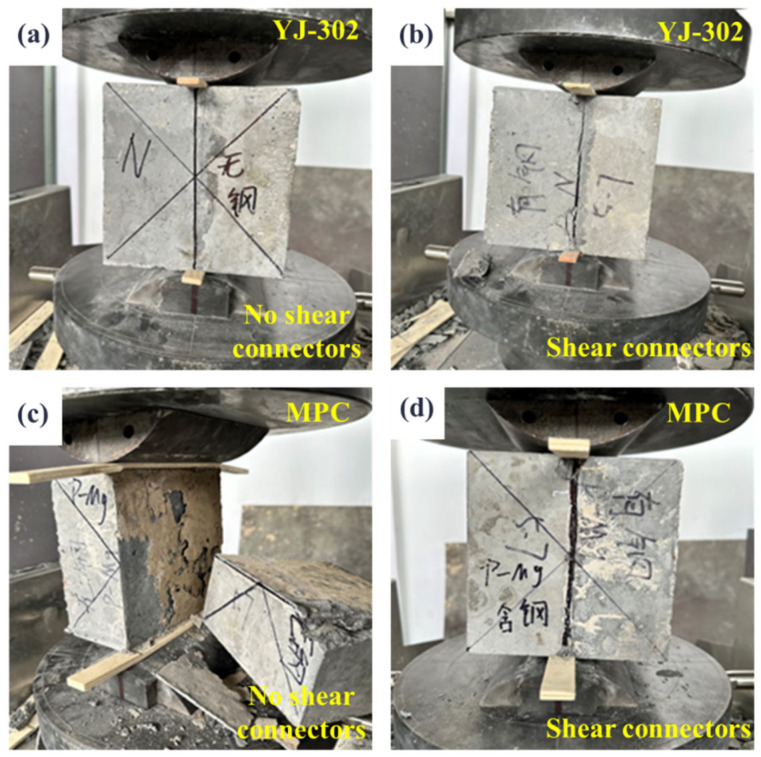
Splitting tensile failure of composite structures with different IBAs. (**a**) YJ-302 and no HSSK; (**b**) YJ-302 + HSSK; (**c**) MPC and no HSSK; (**d**) MPC + HSSK.

Figure 16 illustrates the failure modes of specimens with different bonding agents in the splitting tensile test. As shown in Figure 16b, specimens with horseshoe-shaped shear key reinforcement and YJ-302 interface bonding agent retain a certain degree of integrity after splitting. In contrast, specimens coated solely with MPC (Figure 16c) exhibit direct splitting failure at the interface between the pavement layer concrete and the beam body concrete, with visible MPC residue on the splitting surface, indicating that the concrete layers did not bear the tensile load.

#### 3.3.3. Microstructure

**Figure 17:**
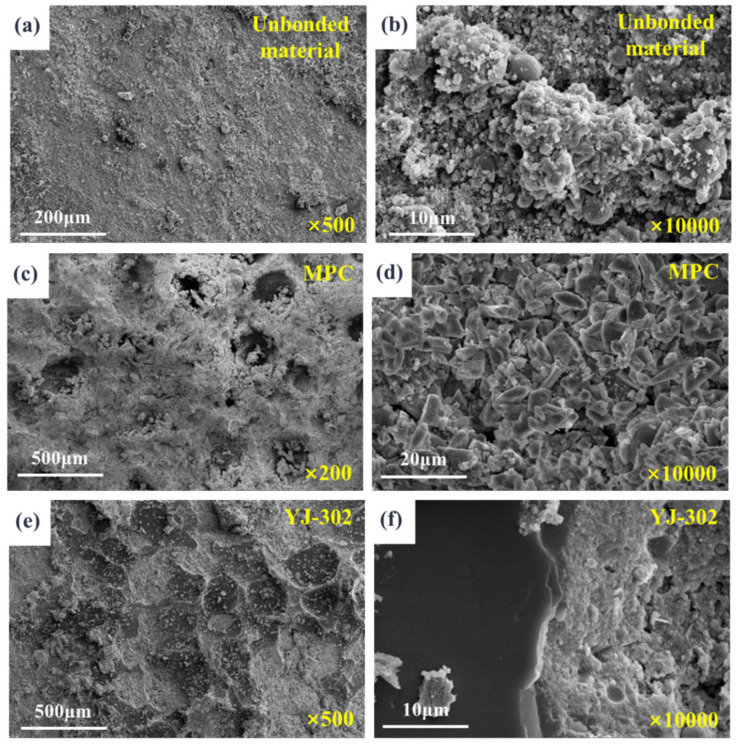
Microstructure of joint surfaces with different binders. (**a**) no bonding materials + 500×; (**b**) no bonding materials + 10,000×; (**c**) MPC + 500×; (**d**) MPC + 10,000×; (**e**) YJ-302 + 500×; (f) YJ-302 + 10,000×.

Figure 17 presents the microstructures of the interfaces in composite systems with different bonding agents. The microstructure of concrete without a bonding agent is relatively dense, and high-magnification images reveal that the primary components are hydration products of cement mortar. In contrast, the microstructure of MPC exhibits numerous pores, and high-magnification images show that the hydration products of MPC have poor cohesion and many voids. Therefore, the application of MPC weakens the mechanical properties of the composite system. The microstructure of the interface coated with the YJ-302 interface bonding agent clearly shows an alternating pattern of the bonding agent and concrete at the microscopic level, possibly due to the tensile failure of the bonding agent during the splitting tensile test. The bonding agent has a highly dense microstructure, providing excellent anchorage points for the interface between the beam body concrete and the pavement layer concrete, thereby enhancing the overall mechanical properties of the composite system.

## 4. Conclusions

In this study, a novel structural form for bridge deck pavement composite systems is developed, eliminating the traditional steel mesh reinforcement and utilizing horseshoe-shaped shear connectors to link the beam body concrete with the pavement layer concrete. By investigating the influence of parameters such as horseshoe-shaped shear connectors, the strength grade and fiber type of the pavement layer concrete, as well as the application of different interfacial bonding agents at the connection surface of the composite system, the following key conclusions were drawn:

(1) The horseshoe-shaped shear connectors enhance the splitting tensile strength of the composite structural system and contribute to maintaining a certain level of integrity when the system undergoes compressive or tensile failure.
(2) As the strength grade of the steel fiber-reinforced concrete in the pavement layer increases, the compressive strength and splitting tensile strength of the composite system rise initially and then stabilize. A recommended strength grade for the pavement layer concrete within the composite system is C40.
(3) The compressive strength of the pavement layer concrete is maximized when it contains steel fibers, while the splitting tensile strength is maximized with the inclusion of POM fibers. Considering overall mechanical performance, steel fibers are recommended as the fiber type for the pavement layer concrete.

## Figures and Tables

**Table 1:** Cement technical index.

Specific Surface Area (m^2^/kg)	Initial Setting Time (min)	Final Setting Time (min)	Flexural Strength (MPa)		Compressive Strength (MPa)	
			3 d	28 d	3 d	28 d
342	204	299	5.9	8.4	29.5	53.3

**Table 2:** The mix ratio of the test.

	Raw Materials (kg/m^3^)						Fiber/%		Water Reducing Agent/%	IBA
	Cement	Fly Ash	Silica Fume	Sand	Gravel	Water				
0	390	80	0	730	1095	155	-	-	0.50%	-
1	390	100	20	802	1021	169	SF	0.51%	0.65%	-
2	340	100	20	802	1021	169	SF	0.51%	0.60%	-
3	300	100	20	802	1021	169	SF	0.51%	0.50%	-
4	340	100	20	802	1021	169	SF	0.51%	0.60%	MPC
5	340	100	20	802	1021	169	SF	0.51%	0.60%	YJ-302
6	340	100	20	802	1021	169	PP	0.51%	0.65%	-
7	340	100	20	802	1021	169	POM	0.51%	0.60%	-

Note: The superplasticizer dosage is expressed as a percentage of the total cementitious material weight; the fiber dosage is given as a volumetric fraction of the concrete. Group 0 represents the simulated C50 concrete for the beam body; Groups 1 to 7 represent the simulated pavement layer concrete, with Groups 1 to 3 being C50, C40, and C30 pavement layer concretes, respectively. Groups 2, 4, and 5 varied the bonding agent between the beam body and the pavement layer concrete; Groups 2, 6, and 7 varied the fiber type in the pavement layer concrete. Furthermore, Groups 1 to 5 included both specimens without shear keys and specimens with shear keys, while Groups 6 and 7 only included specimens with shear keys.

## Data Availability

The original contributions presented in the study are included in the article, further inquiries can be directed to the corresponding author.
